# *tert*-Butyl (2*S*)-2-{3-[(*R*)-bis­(*tert*-but­oxy­carbon­yl)amino]-2-oxopiperidin-1-yl}-3-methyl­butano­ate^1^

**DOI:** 10.1107/S1600536811043212

**Published:** 2011-10-29

**Authors:** Michael J. Kangas, Frank R. Fronczek, Steven F. Watkins

**Affiliations:** a Department of Chemistry, Louisiana State University, Baton Rouge, LA 70803-1804, USA

## Abstract

The title compound, C_24_H_42_N_2_O_7_, is a chiral lactam-constrained amino acid with a six-membered ring backbone and isopropyl and *tert*-butyl ester side chains. The conformation of the six-membered ring can be described as a half chair, with two CH_2_ C atoms lying 0.443 (1) and −0.310 (1) Å out of the best plane of the other four atoms (mean deviation = 0.042 Å). Both N atoms are *sp* ^2^ hybridized, lying 0.0413 (9) and 0.067 (1) Å out of the planes defined by the three C atoms bonded to them. The absolute configuration was determined, based on resonant scattering of light atoms in Cu *K*α radiation.

## Related literature

For synthesis and chemical inter­est, see: Oguz (2003 ▶); Oguz *et al.* (2001 ▶). For a similar structure, see: Valle *et al.* (1989 ▶). For absolute configuration parameters, see: Hooft *et al.* (2008 ▶).
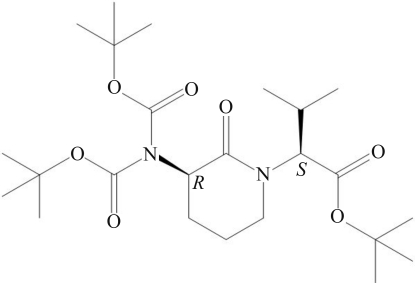


## Experimental

### 

#### Crystal data

C_24_H_42_N_2_O_7_
*M* *_r_* = 470.6
Monoclinic, 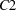

*a* = 27.282 (3) Å
*b* = 9.4315 (10) Å
*c* = 11.5884 (10) Å
β = 110.729 (1)°
*V* = 2788.7 (5) Å^3^
*Z* = 4
Cu *K*α radiation
μ = 0.67 mm^−1^
*T* = 86 K
0.35 × 0.25 × 0.20 mm

#### Data collection

Bruker Kappa APEXII CCD diffractometer
Absorption correction: multi-scan (*SADABS*; Sheldrick, 2004 ▶) *T*_min_ = 0.800, *T*_max_ = 0.878
10823 measured reflections
4844 independent reflections
4782 reflections with *I* > 2σ(*I*)
*R*_int_ = 0.023

#### Refinement

*R*[*F* ^2^ > 2σ(*F* ^2^)] = 0.026
*wR*(*F* ^2^) = 0.071
*S* = 1.01
4844 reflections
310 parameters
1 restraint
H-atom parameters constrained
Δρ_max_ = 0.19 e Å^−3^
Δρ_min_ = −0.12 e Å^−3^
Absolute structure: Flack (1983 ▶), 2174 Friedel pairs
Flack parameter: −0.02 (10)

### 

Data collection: *APEX2* (Bruker, 2006 ▶); cell refinement: *APEX2*; data reduction: *APEX2*; program(s) used to solve structure: *SHELXS97* (Sheldrick, 2008 ▶); program(s) used to refine structure: *SHELXL97* (Sheldrick, 2008 ▶); molecular graphics: *ORTEP-3 for Windows* (Farrugia, 1997 ▶); software used to prepare material for publication: *WinGX* (Farrugia, 1999 ▶).

## Supplementary Material

Crystal structure: contains datablock(s) global, I. DOI: 10.1107/S1600536811043212/pv2462sup1.cif

Structure factors: contains datablock(s) I. DOI: 10.1107/S1600536811043212/pv2462Isup2.hkl

Additional supplementary materials:  crystallographic information; 3D view; checkCIF report

## Figures and Tables

**Fig. 1.**
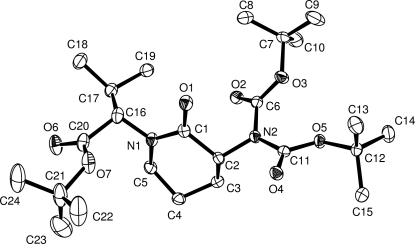
View of the title molecule (50% probability displacement ellipsoids); H atoms are not shown for clarity.

## supplementary crystallographic information

### Comment

Title compound is a conformationally constrained amino acid analog which was designed as part of a study to understand the factors promoting β-sheet formation in brain-degenerative diseases such as Alzheimer's disease, Creutzfeldt-Jacob disease and bovine spongiform encephalopathy (Oguz *et al.*, 2001).

In the title molecule (Fig. 1), the central six-membered ring adopts a conformation close to the C_2_ half chair, with the diad axis bisecting the N1–C1 and C3–C4 bonds. Atoms C1, C2, C5 and N1 are coplanar to within a mean deviation 0.042 Å (maximum 0.0588 (10) Å for N1), the other two atoms lying alternately above and below this plane, C3 by -0.310 (1) and C4 by 0.443 (1) Å. The C5—N1—C1—C2 torsion angle, which would be zero for an ideal half chair, is -13.36 (18)°. This conformation is similar to that seen in a similar lactam-restricted analog of Boc-*L*-Pro-*L*-Leu-Gly-NH_2_, which has torsion angle somewhat closer to zero, 5.6 (11)° and smaller mean deviation for these four atoms, 0.014 Å (Valle *et al.*, 1989).

Both N atoms are *sp*^2^ hybrids, with N1 lying only 0.067 (1) Å from the plane defined by C1, C5 and C16, and N2 lying 0.0413 (9) Å from the plane defined by C2, C6 and C11.

The absolute configuration based on the Flack (1983) parameter *x* = -0.02 (10), the Hooft parameter *y* = -0.05 (5), and the Hooft P2(true) value of 1.000 (Hooft *et al.*, 2008) agrees with that of the starting materials.

### Experimental

The synthesis of the title compound is detailed by Oguz (2003), who prepared a suitable single-crystal by recrystallization from hexanes.

### Refinement

Hydrogen atoms were located from difference maps and included in the refinement in riding mode with C—H distances = 0.98 - 1.00 Å and *U*_iso_(H) = 1.5 *U*_eq_(methyl C) or 1.2 *U*_eq_(non-methyl C). Refinement of the Flack (1983) parameter was used to determine the absolute configurations of the two asymmetric centers in the molecule.

### Crystal data

**Table:**

C_24_H_42_N_2_O_7_	*F*(000) = 1024
*M**_r_* = 470.6	*D*_x_ = 1.121 Mg m^−^^3^
Monoclinic, *C*2	Cu *K*α radiation, λ = 1.54178 Å
Hall symbol: C 2y	Cell parameters from 8306 reflections
*a* = 27.282 (3) Å	θ = 3.5–67.8°
*b* = 9.4315 (10) Å	µ = 0.67 mm^−^^1^
*c* = 11.5884 (10) Å	*T* = 86 K
β = 110.729 (1)°	Fragment, colourless
*V* = 2788.7 (5) Å^3^	0.35 × 0.25 × 0.20 mm
*Z* = 4	

### Data collection

**Table:**

Bruker Kappa APEXII CCD diffractometer	4844 independent reflections
Radiation source: fine-focus sealed tube	4782 reflections with *I* > 2σ(*I*)
graphite	*R*_int_ = 0.023
φ and ω scans	θ_max_ = 68.2°, θ_min_ = 3.5°
Absorption correction: multi-scan (*SADABS*; Sheldrick, 2004)	*h* = −32→32
*T*_min_ = 0.800, *T*_max_ = 0.878	*k* = −10→11
10823 measured reflections	*l* = −13→13

### Refinement

**Table:**

Refinement on *F*^2^	Hydrogen site location: inferred from neighbouring sites
Least-squares matrix: full	H-atom parameters constrained
*R*[*F*^2^ > 2σ(*F*^2^)] = 0.026	*w* = 1/[σ^2^(*F*_o_^2^) + (0.0441*P*)^2^ + 0.6036*P*] where *P* = (*F*_o_^2^ + 2*F*_c_^2^)/3
*wR*(*F*^2^) = 0.071	(Δ/σ)_max_ = 0.001
*S* = 1.01	Δρ_max_ = 0.19 e Å^−^^3^
4844 reflections	Δρ_min_ = −0.12 e Å^−^^3^
310 parameters	Extinction correction: *SHELXS97* (Sheldrick, 2008), Fc^*^=kFc[1+0.001xFc^2^λ^3^/sin(2θ)]^-1/4^
1 restraint	Extinction coefficient: 0.00066 (8)
0 constraints	Absolute structure: Flack (1983), 2174 Friedel pairs
Primary atom site location: structure-invariant direct methods	Flack parameter: −0.02 (10)
Secondary atom site location: difference Fourier map	

### Special details

**Table:**

Geometry. All e.s.d.'s (except the e.s.d. in the dihedral angle between two l.s. planes) are estimated using the full covariance matrix. The cell e.s.d.'s are taken into account individually in the estimation of e.s.d.'s in distances, angles and torsion angles; correlations between e.s.d.'s in cell parameters are only used when they are defined by crystal symmetry. An approximate (isotropic) treatment of cell e.s.d.'s is used for estimating e.s.d.'s involving l.s. planes.

### Fractional atomic coordinates and isotropic or equivalent isotropic displacement parameters (Å^2^)

**Table:**

	*x*	*y*	*z*	*U*_iso_*/*U*_eq_	
C1	0.29466 (4)	0.36747 (13)	0.72598 (11)	0.0195 (2)	
C2	0.24099 (4)	0.41656 (13)	0.63587 (11)	0.0194 (2)	
H2	0.2438	0.5214	0.6267	0.023*	
C3	0.22473 (5)	0.35489 (14)	0.50640 (11)	0.0224 (3)	
H3A	0.194	0.4069	0.4504	0.027*	
H3B	0.2149	0.254	0.5075	0.027*	
C4	0.27044 (5)	0.36775 (14)	0.46077 (11)	0.0225 (3)	
H4A	0.26	0.3321	0.3751	0.027*	
H4B	0.2809	0.4684	0.4616	0.027*	
C5	0.31600 (5)	0.28143 (14)	0.54486 (11)	0.0221 (3)	
H5A	0.307	0.1794	0.5335	0.026*	
H5B	0.3471	0.2973	0.5212	0.026*	
C6	0.20105 (4)	0.26219 (13)	0.74681 (11)	0.0191 (2)	
C7	0.18475 (5)	0.14657 (13)	0.91793 (11)	0.0236 (3)	
C8	0.24179 (5)	0.11115 (18)	0.98719 (13)	0.0343 (3)	
H8A	0.258	0.0784	0.9288	0.051*	
H8B	0.2442	0.0363	1.0476	0.051*	
H8C	0.2601	0.1959	1.03	0.051*	
C9	0.15900 (7)	0.20383 (18)	1.00423 (15)	0.0412 (4)	
H9A	0.1789	0.2857	1.049	0.062*	
H9B	0.1584	0.1299	1.0632	0.062*	
H9C	0.123	0.233	0.9564	0.062*	
C10	0.15454 (6)	0.02203 (16)	0.84575 (14)	0.0359 (3)	
H10A	0.1191	0.0526	0.795	0.054*	
H10B	0.1524	−0.0522	0.9029	0.054*	
H10C	0.1725	−0.0153	0.7923	0.054*	
C11	0.16978 (5)	0.51181 (13)	0.69474 (10)	0.0183 (2)	
C12	0.08595 (4)	0.57415 (14)	0.71249 (11)	0.0208 (2)	
C13	0.10986 (6)	0.63856 (16)	0.83951 (13)	0.0310 (3)	
H13A	0.1201	0.5629	0.9014	0.046*	
H13B	0.0841	0.7004	0.8556	0.046*	
H13C	0.1408	0.6942	0.8439	0.046*	
C14	0.03931 (5)	0.48195 (15)	0.70301 (14)	0.0295 (3)	
H14A	0.0269	0.4342	0.6227	0.044*	
H14B	0.0112	0.5411	0.7112	0.044*	
H14C	0.0497	0.4109	0.769	0.044*	
C15	0.07098 (5)	0.68538 (16)	0.61068 (13)	0.0294 (3)	
H15A	0.1005	0.7501	0.6232	0.044*	
H15B	0.0406	0.7391	0.6129	0.044*	
H15C	0.0622	0.6385	0.5304	0.044*	
C16	0.38183 (5)	0.27894 (15)	0.75821 (11)	0.0241 (3)	
H16	0.3863	0.3166	0.842	0.029*	
C17	0.39093 (5)	0.11852 (15)	0.77060 (11)	0.0243 (3)	
H17	0.3935	0.0812	0.6922	0.029*	
C18	0.44260 (5)	0.08832 (18)	0.87695 (12)	0.0339 (3)	
H18A	0.4404	0.1243	0.9543	0.051*	
H18B	0.4715	0.1355	0.8606	0.051*	
H18C	0.4489	−0.0142	0.8838	0.051*	
C19	0.34592 (5)	0.04375 (15)	0.79477 (12)	0.0273 (3)	
H19A	0.3531	−0.0582	0.8044	0.041*	
H19B	0.3132	0.06	0.7251	0.041*	
H19C	0.3425	0.0815	0.8704	0.041*	
C20	0.42136 (5)	0.35571 (15)	0.71361 (13)	0.0266 (3)	
C21	0.44367 (6)	0.59566 (17)	0.66729 (18)	0.0416 (4)	
C22	0.42311 (7)	0.73765 (19)	0.6920 (3)	0.0610 (6)	
H22A	0.3851	0.7418	0.6487	0.091*	
H22B	0.4399	0.8141	0.6623	0.091*	
H22C	0.431	0.7487	0.7808	0.091*	
C23	0.42947 (9)	0.5699 (2)	0.5302 (2)	0.0585 (5)	
H23A	0.4446	0.4798	0.5173	0.088*	
H23B	0.4433	0.6473	0.4942	0.088*	
H23C	0.3913	0.5662	0.4905	0.088*	
C24	0.50190 (6)	0.5826 (2)	0.7382 (2)	0.0567 (5)	
H24A	0.5086	0.5904	0.8268	0.085*	
H24B	0.5205	0.6586	0.713	0.085*	
H24C	0.5143	0.4904	0.7207	0.085*	
N1	0.32921 (4)	0.31854 (11)	0.67548 (9)	0.0202 (2)	
N2	0.20095 (4)	0.39526 (11)	0.69179 (9)	0.0193 (2)	
O1	0.30559 (3)	0.38315 (10)	0.83680 (8)	0.0241 (2)	
O2	0.21970 (3)	0.15970 (9)	0.71609 (8)	0.02276 (19)	
O3	0.18130 (4)	0.27044 (9)	0.83558 (8)	0.02354 (19)	
O4	0.18374 (3)	0.63235 (9)	0.69062 (8)	0.02172 (18)	
O5	0.12315 (3)	0.46991 (9)	0.69416 (8)	0.02019 (18)	
O6	0.45332 (4)	0.29793 (11)	0.68025 (10)	0.0334 (2)	
O7	0.41427 (4)	0.49542 (10)	0.71647 (10)	0.0339 (2)	

### Atomic displacement parameters (Å^2^)

**Table:**

	*U*^11^	*U*^22^	*U*^33^	*U*^12^	*U*^13^	*U*^23^
C1	0.0190 (5)	0.0172 (6)	0.0226 (6)	−0.0022 (5)	0.0079 (5)	−0.0031 (5)
C2	0.0191 (5)	0.0204 (6)	0.0220 (6)	0.0017 (5)	0.0114 (5)	0.0025 (5)
C3	0.0210 (6)	0.0251 (6)	0.0202 (6)	0.0024 (5)	0.0062 (5)	0.0028 (5)
C4	0.0256 (6)	0.0246 (6)	0.0189 (6)	0.0019 (5)	0.0099 (5)	0.0014 (5)
C5	0.0238 (6)	0.0250 (6)	0.0201 (6)	0.0014 (5)	0.0112 (5)	−0.0030 (5)
C6	0.0170 (5)	0.0195 (6)	0.0201 (6)	0.0002 (4)	0.0059 (4)	0.0016 (5)
C7	0.0273 (6)	0.0211 (6)	0.0252 (6)	0.0021 (5)	0.0130 (5)	0.0082 (5)
C8	0.0304 (7)	0.0421 (8)	0.0278 (7)	0.0013 (6)	0.0072 (6)	0.0126 (6)
C9	0.0626 (10)	0.0347 (8)	0.0419 (8)	0.0146 (7)	0.0380 (8)	0.0147 (7)
C10	0.0350 (7)	0.0340 (8)	0.0393 (8)	−0.0104 (6)	0.0140 (6)	0.0054 (6)
C11	0.0189 (5)	0.0210 (6)	0.0154 (5)	0.0016 (5)	0.0066 (4)	0.0016 (4)
C12	0.0190 (6)	0.0217 (6)	0.0242 (6)	0.0050 (5)	0.0107 (5)	0.0023 (5)
C13	0.0322 (7)	0.0346 (7)	0.0312 (7)	0.0003 (6)	0.0175 (6)	−0.0046 (6)
C14	0.0232 (6)	0.0280 (7)	0.0417 (8)	0.0010 (6)	0.0171 (5)	0.0017 (6)
C15	0.0244 (6)	0.0317 (7)	0.0348 (7)	0.0088 (6)	0.0139 (5)	0.0106 (6)
C16	0.0176 (6)	0.0314 (7)	0.0226 (6)	0.0003 (5)	0.0064 (5)	−0.0077 (5)
C17	0.0219 (6)	0.0315 (7)	0.0194 (6)	0.0051 (5)	0.0071 (5)	−0.0030 (5)
C18	0.0242 (6)	0.0494 (9)	0.0255 (7)	0.0091 (6)	0.0055 (5)	−0.0005 (6)
C19	0.0268 (6)	0.0316 (7)	0.0237 (6)	0.0050 (5)	0.0093 (5)	0.0027 (5)
C20	0.0177 (6)	0.0295 (7)	0.0300 (6)	−0.0012 (5)	0.0055 (5)	−0.0103 (5)
C21	0.0267 (7)	0.0263 (8)	0.0749 (11)	−0.0062 (6)	0.0220 (7)	−0.0076 (7)
C22	0.0371 (9)	0.0299 (9)	0.1201 (19)	−0.0021 (7)	0.0331 (11)	−0.0089 (10)
C23	0.0633 (11)	0.0450 (10)	0.0737 (13)	−0.0162 (9)	0.0321 (10)	0.0036 (10)
C24	0.0276 (8)	0.0362 (9)	0.1048 (16)	−0.0080 (7)	0.0215 (9)	−0.0115 (10)
N1	0.0176 (5)	0.0245 (5)	0.0188 (5)	0.0002 (4)	0.0067 (4)	−0.0046 (4)
N2	0.0185 (5)	0.0181 (5)	0.0239 (5)	0.0023 (4)	0.0109 (4)	0.0029 (4)
O1	0.0227 (4)	0.0302 (5)	0.0204 (4)	0.0020 (4)	0.0088 (3)	−0.0057 (4)
O2	0.0266 (4)	0.0188 (4)	0.0252 (4)	0.0030 (3)	0.0122 (3)	0.0011 (3)
O3	0.0299 (4)	0.0195 (4)	0.0266 (4)	0.0046 (4)	0.0167 (4)	0.0051 (4)
O4	0.0231 (4)	0.0181 (4)	0.0272 (4)	0.0004 (3)	0.0129 (3)	0.0020 (3)
O5	0.0181 (4)	0.0183 (4)	0.0266 (4)	0.0021 (3)	0.0110 (3)	0.0010 (3)
O6	0.0260 (5)	0.0315 (5)	0.0491 (6)	−0.0011 (4)	0.0210 (4)	−0.0096 (4)
O7	0.0236 (5)	0.0268 (5)	0.0550 (6)	−0.0036 (4)	0.0184 (4)	−0.0112 (5)

### Geometric parameters (Å, °)

**Table:**

C1—O1	1.2202 (15)	C13—H13A	0.98
C1—N1	1.3545 (16)	C13—H13B	0.98
C1—C2	1.5382 (15)	C13—H13C	0.98
C2—N2	1.4679 (15)	C14—H14A	0.98
C2—C3	1.5215 (17)	C14—H14B	0.98
C2—H2	1	C14—H14C	0.98
C3—C4	1.5220 (16)	C15—H15A	0.98
C3—H3A	0.99	C15—H15B	0.98
C3—H3B	0.99	C15—H15C	0.98
C4—C5	1.5165 (17)	C16—N1	1.4646 (15)
C4—H4A	0.99	C16—C17	1.5317 (19)
C4—H4B	0.99	C16—C20	1.5327 (19)
C5—N1	1.4688 (15)	C16—H16	1
C5—H5A	0.99	C17—C19	1.5254 (18)
C5—H5B	0.99	C17—C18	1.5353 (17)
C6—O2	1.2035 (15)	C17—H17	1
C6—O3	1.3217 (15)	C18—H18A	0.98
C6—N2	1.4074 (16)	C18—H18B	0.98
C7—O3	1.4902 (14)	C18—H18C	0.98
C7—C10	1.507 (2)	C19—H19A	0.98
C7—C9	1.5107 (19)	C19—H19B	0.98
C7—C8	1.5145 (18)	C19—H19C	0.98
C8—H8A	0.98	C20—O6	1.2021 (17)
C8—H8B	0.98	C20—O7	1.3339 (18)
C8—H8C	0.98	C21—O7	1.4785 (19)
C9—H9A	0.98	C21—C24	1.513 (2)
C9—H9B	0.98	C21—C23	1.515 (3)
C9—H9C	0.98	C21—C22	1.518 (2)
C10—H10A	0.98	C22—H22A	0.98
C10—H10B	0.98	C22—H22B	0.98
C10—H10C	0.98	C22—H22C	0.98
C11—O4	1.2052 (15)	C23—H23A	0.98
C11—O5	1.3298 (15)	C23—H23B	0.98
C11—N2	1.3976 (16)	C23—H23C	0.98
C12—O5	1.4813 (14)	C24—H24A	0.98
C12—C13	1.5105 (18)	C24—H24B	0.98
C12—C14	1.5127 (17)	C24—H24C	0.98
C12—C15	1.5224 (17)		
			
O1—C1—N1	123.21 (11)	H14A—C14—H14B	109.5
O1—C1—C2	119.83 (11)	C12—C14—H14C	109.5
N1—C1—C2	116.74 (10)	H14A—C14—H14C	109.5
N2—C2—C3	112.35 (10)	H14B—C14—H14C	109.5
N2—C2—C1	109.72 (9)	C12—C15—H15A	109.5
C3—C2—C1	115.44 (10)	C12—C15—H15B	109.5
N2—C2—H2	106.2	H15A—C15—H15B	109.5
C3—C2—H2	106.2	C12—C15—H15C	109.5
C1—C2—H2	106.2	H15A—C15—H15C	109.5
C2—C3—C4	108.87 (10)	H15B—C15—H15C	109.5
C2—C3—H3A	109.9	N1—C16—C17	113.71 (10)
C4—C3—H3A	109.9	N1—C16—C20	107.61 (11)
C2—C3—H3B	109.9	C17—C16—C20	112.84 (10)
C4—C3—H3B	109.9	N1—C16—H16	107.5
H3A—C3—H3B	108.3	C17—C16—H16	107.5
C5—C4—C3	108.66 (10)	C20—C16—H16	107.5
C5—C4—H4A	110	C19—C17—C16	111.07 (10)
C3—C4—H4A	110	C19—C17—C18	109.69 (11)
C5—C4—H4B	110	C16—C17—C18	109.32 (11)
C3—C4—H4B	110	C19—C17—H17	108.9
H4A—C4—H4B	108.3	C16—C17—H17	108.9
N1—C5—C4	112.37 (10)	C18—C17—H17	108.9
N1—C5—H5A	109.1	C17—C18—H18A	109.5
C4—C5—H5A	109.1	C17—C18—H18B	109.5
N1—C5—H5B	109.1	H18A—C18—H18B	109.5
C4—C5—H5B	109.1	C17—C18—H18C	109.5
H5A—C5—H5B	107.9	H18A—C18—H18C	109.5
O2—C6—O3	127.53 (11)	H18B—C18—H18C	109.5
O2—C6—N2	121.03 (11)	C17—C19—H19A	109.5
O3—C6—N2	111.35 (10)	C17—C19—H19B	109.5
O3—C7—C10	110.94 (10)	H19A—C19—H19B	109.5
O3—C7—C9	101.83 (10)	C17—C19—H19C	109.5
C10—C7—C9	110.80 (12)	H19A—C19—H19C	109.5
O3—C7—C8	109.41 (10)	H19B—C19—H19C	109.5
C10—C7—C8	112.32 (12)	O6—C20—O7	125.73 (14)
C9—C7—C8	111.06 (12)	O6—C20—C16	124.84 (13)
C7—C8—H8A	109.5	O7—C20—C16	109.43 (11)
C7—C8—H8B	109.5	O7—C21—C24	110.31 (15)
H8A—C8—H8B	109.5	O7—C21—C23	109.23 (13)
C7—C8—H8C	109.5	C24—C21—C23	112.71 (16)
H8A—C8—H8C	109.5	O7—C21—C22	101.79 (13)
H8B—C8—H8C	109.5	C24—C21—C22	110.88 (14)
C7—C9—H9A	109.5	C23—C21—C22	111.37 (18)
C7—C9—H9B	109.5	C21—C22—H22A	109.5
H9A—C9—H9B	109.5	C21—C22—H22B	109.5
C7—C9—H9C	109.5	H22A—C22—H22B	109.5
H9A—C9—H9C	109.5	C21—C22—H22C	109.5
H9B—C9—H9C	109.5	H22A—C22—H22C	109.5
C7—C10—H10A	109.5	H22B—C22—H22C	109.5
C7—C10—H10B	109.5	C21—C23—H23A	109.5
H10A—C10—H10B	109.5	C21—C23—H23B	109.5
C7—C10—H10C	109.5	H23A—C23—H23B	109.5
H10A—C10—H10C	109.5	C21—C23—H23C	109.5
H10B—C10—H10C	109.5	H23A—C23—H23C	109.5
O4—C11—O5	126.53 (11)	H23B—C23—H23C	109.5
O4—C11—N2	122.50 (11)	C21—C24—H24A	109.5
O5—C11—N2	110.82 (10)	C21—C24—H24B	109.5
O5—C12—C13	108.98 (10)	H24A—C24—H24B	109.5
O5—C12—C14	102.08 (10)	C21—C24—H24C	109.5
C13—C12—C14	111.34 (11)	H24A—C24—H24C	109.5
O5—C12—C15	110.83 (10)	H24B—C24—H24C	109.5
C13—C12—C15	112.52 (12)	C1—N1—C16	118.35 (10)
C14—C12—C15	110.60 (11)	C1—N1—C5	124.89 (10)
C12—C13—H13A	109.5	C16—N1—C5	116.10 (10)
C12—C13—H13B	109.5	C11—N2—C6	126.44 (10)
H13A—C13—H13B	109.5	C11—N2—C2	117.23 (10)
C12—C13—H13C	109.5	C6—N2—C2	116.08 (10)
H13A—C13—H13C	109.5	C6—O3—C7	120.10 (10)
H13B—C13—H13C	109.5	C11—O5—C12	120.31 (9)
C12—C14—H14A	109.5	C20—O7—C21	121.25 (11)
C12—C14—H14B	109.5		
			
O1—C1—C2—N2	−34.52 (15)	O5—C11—N2—C6	−33.73 (15)
N1—C1—C2—N2	150.65 (10)	O4—C11—N2—C2	−23.56 (16)
O1—C1—C2—C3	−162.67 (11)	O5—C11—N2—C2	152.30 (10)
N1—C1—C2—C3	22.50 (16)	O2—C6—N2—C11	162.50 (11)
N2—C2—C3—C4	−174.17 (10)	O3—C6—N2—C11	−20.70 (16)
C1—C2—C3—C4	−47.33 (14)	O2—C6—N2—C2	−23.47 (16)
C2—C3—C4—C5	62.18 (13)	O3—C6—N2—C2	153.33 (10)
C3—C4—C5—N1	−52.90 (14)	C3—C2—N2—C11	−102.88 (12)
N1—C16—C17—C19	−47.91 (14)	C1—C2—N2—C11	127.27 (11)
C20—C16—C17—C19	−170.83 (10)	C3—C2—N2—C6	82.52 (13)
N1—C16—C17—C18	−169.09 (10)	C1—C2—N2—C6	−47.33 (14)
C20—C16—C17—C18	67.99 (14)	O2—C6—O3—C7	5.36 (18)
N1—C16—C20—O6	−119.62 (14)	N2—C6—O3—C7	−171.17 (9)
C17—C16—C20—O6	6.64 (18)	C10—C7—O3—C6	−63.25 (15)
N1—C16—C20—O7	59.70 (13)	C9—C7—O3—C6	178.81 (12)
C17—C16—C20—O7	−174.04 (10)	C8—C7—O3—C6	61.23 (15)
O1—C1—N1—C16	1.70 (18)	O4—C11—O5—C12	−11.94 (17)
C2—C1—N1—C16	176.34 (11)	N2—C11—O5—C12	172.41 (9)
O1—C1—N1—C5	172.01 (12)	C13—C12—O5—C11	−62.98 (14)
C2—C1—N1—C5	−13.36 (18)	C14—C12—O5—C11	179.17 (10)
C17—C16—N1—C1	105.82 (13)	C15—C12—O5—C11	61.37 (13)
C20—C16—N1—C1	−128.44 (12)	O6—C20—O7—C21	5.1 (2)
C17—C16—N1—C5	−65.34 (14)	C16—C20—O7—C21	−174.19 (12)
C20—C16—N1—C5	60.40 (14)	C24—C21—O7—C20	−61.66 (19)
C4—C5—N1—C1	29.46 (17)	C23—C21—O7—C20	62.76 (18)
C4—C5—N1—C16	−160.04 (11)	C22—C21—O7—C20	−179.40 (14)
O4—C11—N2—C6	150.41 (12)		
